# T-Cell Receptor Variable β Domains Rigidify During Affinity Maturation

**DOI:** 10.1038/s41598-020-61433-0

**Published:** 2020-03-11

**Authors:** Monica L. Fernández-Quintero, Clarissa A. Seidler, Klaus R. Liedl

**Affiliations:** Institute of General, Inorganic and Theoretical Chemistry, and Center for Molecular Biosciences Innsbruck (CMBI), University of Innsbruck, Innrain 80-82, A-6020 Innsbruck, Austria

**Keywords:** Computational biology and bioinformatics, Computational models, Protein analysis, Immunology, Antigen processing and presentation, Biophysics, Molecular biophysics, Structural biology, Molecular modelling

## Abstract

We investigated T-cell receptor variable β chains binding to the superantigen staphylococcal enterotoxin C3 (SEC 3) with structure information in different stages of affinity maturation. Metadynamics in combination with molecular dynamics simulations allow to access the micro-to-millisecond timescale and reveal a strong effect of energetically significant mutations on the flexibility of the antigen-binding site. The observed changes in dynamics of the complementarity determining region (CDR) loops, especially the CDR 2, and HV 4 loop on this specific pathway of affinity maturation are reflected in their structural diversity, thermodynamics of conformations and kinetics of structural transitions. In addition, this affinity maturation pathway follows the concept of conformational selection, because even without the presence of the antigen the binding competent state is present in this pre-existing ensemble of conformations. In all stages of this affinity maturation process we observe a link between specificity and reduced flexibility.

## Introduction

T-cells play an important signaling role in the adaptive immune system and are activated upon antigen recognition by their T-cell receptors (TCRs)^1^. TCRs are expressed on two types of cells, cytotoxic T-cells and helper T-cells. TCRs consist of an α and ß chain, which can be divided into a constant and a variable domain, homologous to the immunoglobins^2^. TCR α chains are made from V and J genes, analogous to light chains in antibodies, while TCR ß chains, same as for antibody heavy chains, are assembled from the V, D and J genes^3–6^. Sequence and structural diversity are concentrated on six hypervariable loops, also known as complementarity determining regions (CDRs), each three loops on the α and ß variable domains, which are critical for antigen recognition^7–9^. TCR CDR 1 and CDR 2 loops show interactions with the major histocompatibility complexes (MHC I and MHC II), while CDR 3 has contacts with peptide antigens. Similar to antibodies the TCR CDRs can adopt well-characterized canonical conformations which facilitate reliable structure prediction based on sequence information^3^. Still a clear characterization of structure and dynamics is essential to understand the antigen-binding process, the involved conformational changes and the associated biological implications^10^. Various studies focused already on understanding the evolution of an antibody-antigen interface during affinity maturation^11–15^. However, elucidating the physicochemical principles that are involved in protein-protein interactions, would improve the understanding of biological systems^16^. Structural basis of affinity maturation has been established for an *in vitro* designed protein-protein interaction system which provides with the help of strong structural experimental data molecular snapshots of the affinity maturation process and the involved remodeling of the protein interface^17,18^. The energetic contributions of individual amino acid residues to the complex have been investigated to get a better understanding of intramolecular cooperativity and to be able to predict specificity^17,18^. The correlation between enhanced specificity and rigidification is often discussed in terms of conformational selection^19–21^. Promiscuity might arise from a multitude of weakly populated conformations, each of which is able to bind different binding partners. Rigidification shifts the probability toward a small number of states and thereby reduces the amount of possible binding partners^20,22–24^.

We analyzed an affinity maturation pathway of murine TCR Vß variants with structural information for every stage to elucidate the underlying mechanism of the affinity maturation process and to identify differences in their conformational diversity. We focused on the CDR 2 and the HV 4 loop to investigate changes in dynamics as a consequence of point-mutations, because they directly interact with the superantigen staphylococcal enterotoxin C3 (SEC 3) and are the mutation hotspots in this affinity maturation pathway.

## Results

**Figure 1:**
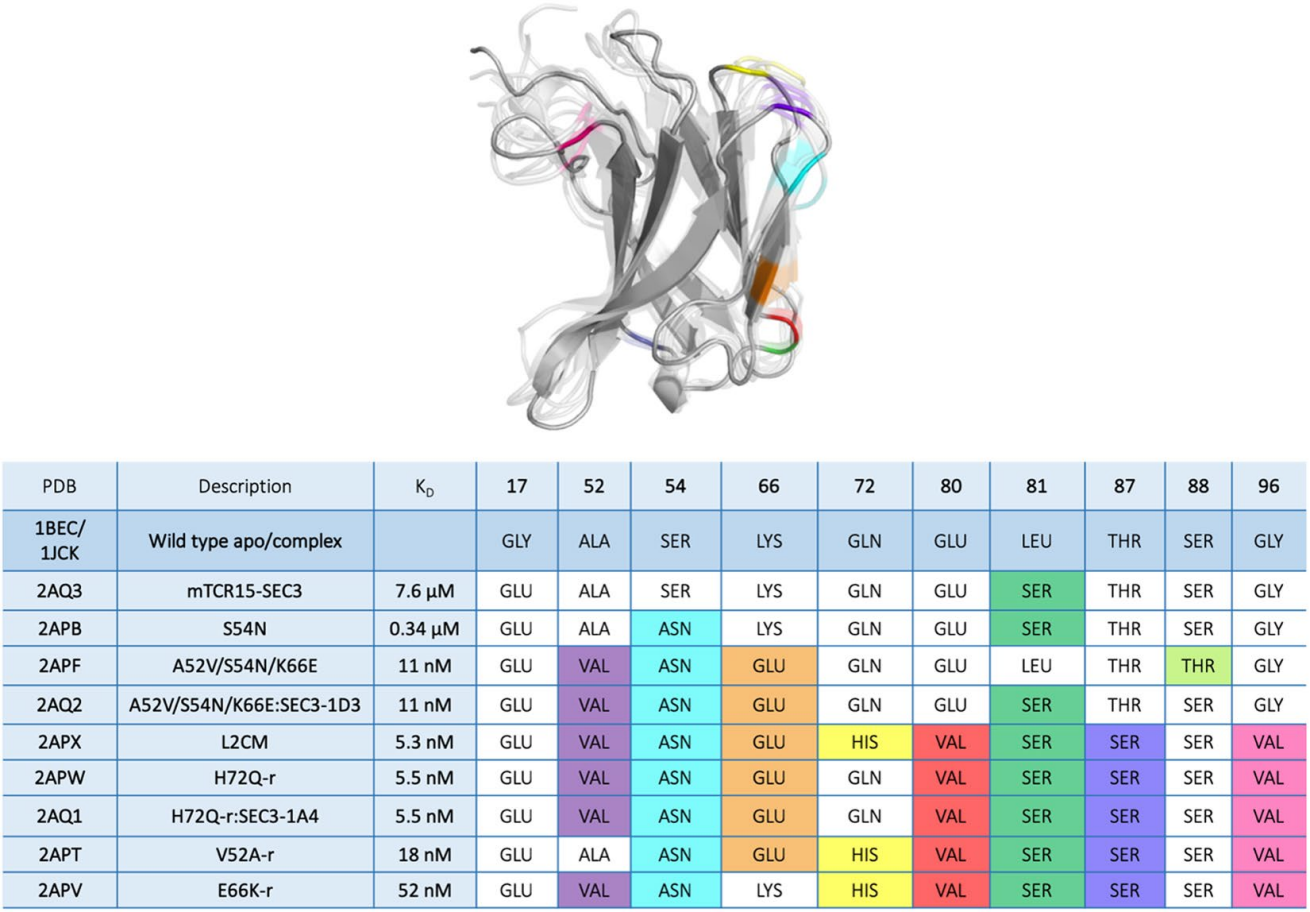
Summary and overview of all available crystal structures in different stages of affinity maturation with the color-coded positions of the point-mutations (residue number and residue type) on a representative TCR Vß variant.

A previous study investigated the cooperative and energetic influence of point-mutations in murine TCR Vß variants^17,18^. Experimental structure information was available for all stages of affinity maturation. Figure 1 summarizes all available crystal structures, all point-mutations and their positions, color coded respectively, on a representative TCR Vß variant structure.

**Figure 2:**
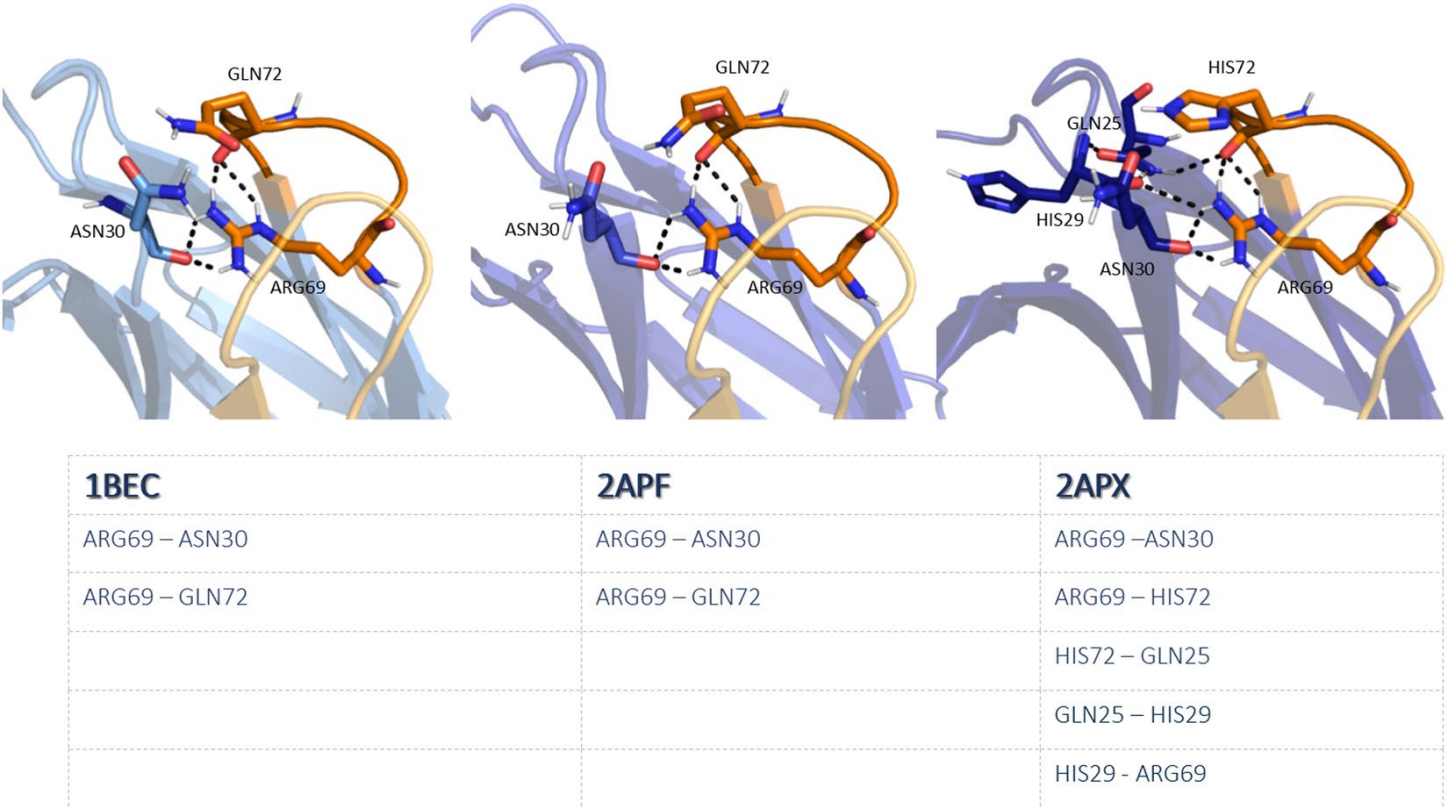
Structural interaction analysis of Vß crystal structures in different stages of affinity maturation. The CDR2 and HV 4 loop are colored yellow and orange, respectively, while in blue the interaction partners in different stages of affinity maturation are illustrated. Strong ionic and hydrogen-bond interactions are present in the X-ray structures with the accession codes 1BEC, 2APF and 2APX.

Figure 2 shows the ionic and hydrogen bond interactions formed in the TCR Vß variants in different stages of affinity maturation. A clear structural stabilization can be observed for the most affinity matured variant compared to the wildtype.

**Figure 3:**
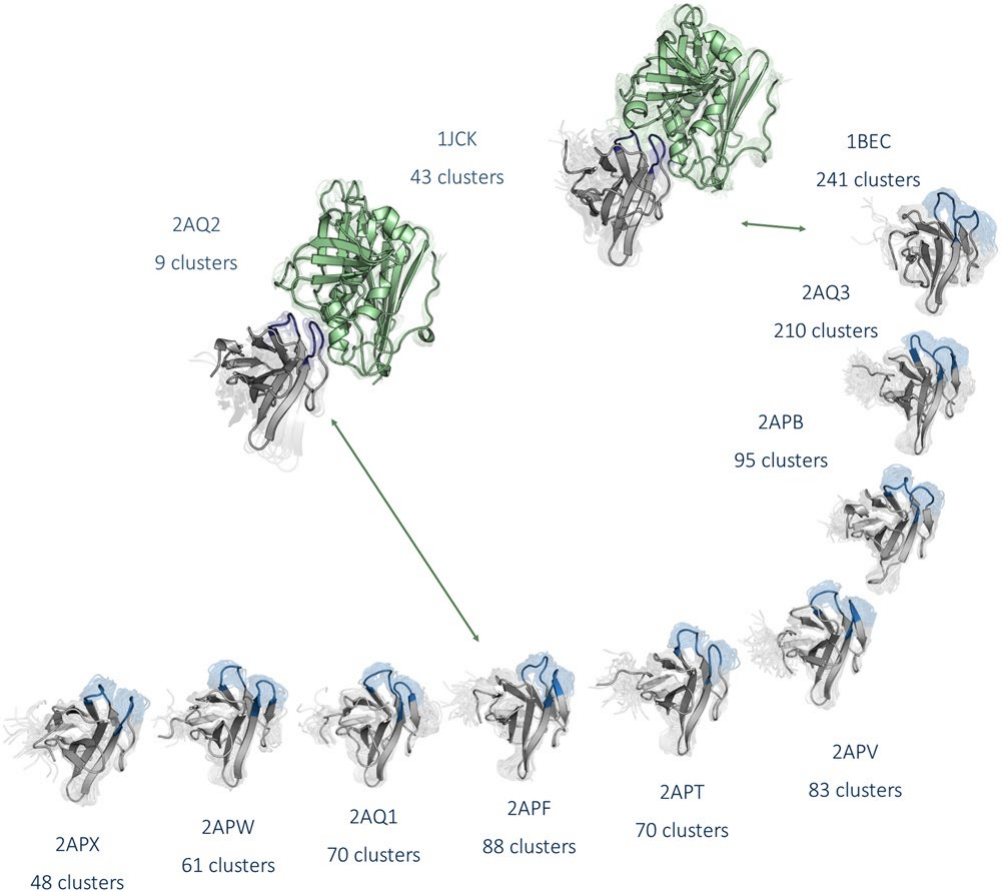
Overview of all analyzed TCR variants with the resulting conformational ensembles of the CDR 2 and the HV 4 loop colored in blue of each 1 µs metadynamics simulations. The diversity of the conformational ensemble is characterized by the number of clusters for each simulation and decreases with higher stages of maturation. The wildtype 1JCK and the affinity matured variant 2AQ2 were additionally simulated with the antigen present to investigate the role of binding to SEC 3 on the observed conformational ensemble. The resulting ensembles clearly follow the paradigm of conformational selection.

As described in the methods section, the metadynamics simulations for each available starting structure was clustered using the same distance cut-off with the aim of having representative structures distributed over the entire sampled conformational space. This strategy resulted in 241 clusters of the CDR 2 and the HV 4 loop for the wildtype TCR Vß variant 1BEC and in 43 clusters for the wildtype TCR Vß 1JCK complexed with SEC 3. The conformational diversity of 1BEC upon binding to SEC 3 (1JCK) in the resulting ensemble is substantially decreased and represents a prime example of conformational selection. The TCR variant 2AQ3 is the most similar to the wildtype and displays a comparable conformational diversity reflected in 210 cluster representatives. Significantly reduced conformational diversity was observed in the most matured TCR Vß variant 2APX which leads to 48 cluster representatives. An overview of the resulting CDR 2 and HV 4 conformational ensembles of the whole affinity maturation pathway is illustrated in Fig. 3. A more detailed perspective on the CDR 2 and HV 4 loop ensembles upon affinity maturation is shown in SI Fig. S1. The variant 2AQ2 was simulated with the antigen SEC 3 present to investigate the influence of the antigen-binding on the observed dynamics. The TCR variant 2APF binds with the same binding affinity as the 2AQ2 variant and is crystallized without SEC 3. By comparing the conformational diversity of 2APF with 2AQ2 the number of accessible CDR 2 and HV 4 loop conformations substantially decreases from 88 to 9 clusters.

**Figure 4:**
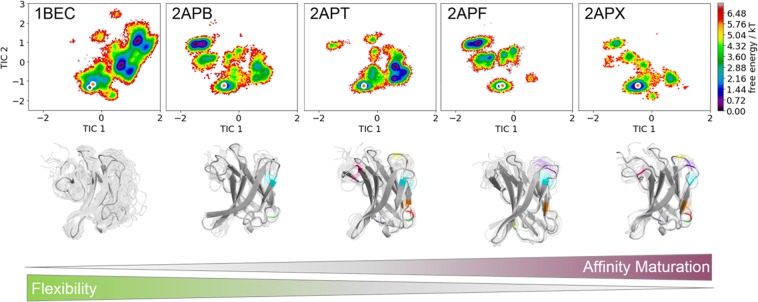
TICA analyses for five TCR Vß variants in different stages of affinity maturation clearly show a significant rigidification of CDR 2 and the HV 4 loop. All TCR Vß variants are projected onto the combined tICA space of 24.1 µs trajectories of 1BEC, 9.5 µs trajectories of 2APB, 7 µs trajectories of 2APT, 8.8 µs trajectories of 2APF and 4.8 µs trajectories of 2APX. The X-ray structures crystallized without antigen are colored in orange, while the X-ray structures crystallized with antigen are colored blue. Both of them are projected into the tICA analyses. Additionally, a graphical representation of the loop flexibility is shown as representative ensemble below the tICA analyses.

Figure 4 shows the combined tICA analyses using the same coordinate system for five TCR Vß variants in different stages of affinity maturation. The tICA space displays conformational shifts on this affinity maturation pathway and a substantial reduction in conformational space. Below the resulting conformational ensembles of the CDR 2 and HV 4 loop are illustrated with the color coded positions of their point mutations.

**Figure 5:**
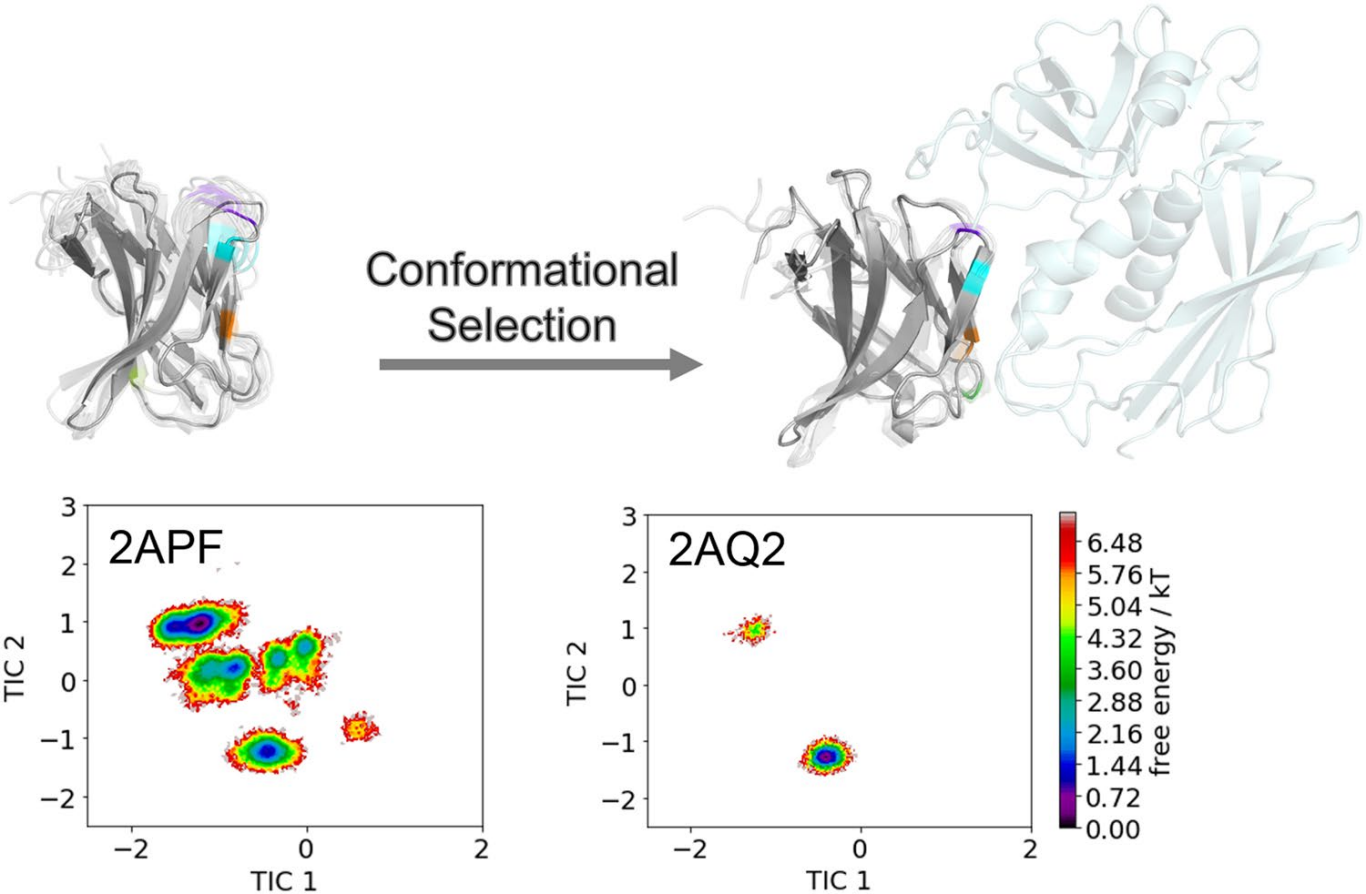
Comparison of the resulting CDR 2 and HV 4 loop ensembles of 8.8 µs 2APF trajectories and 1 µs 2AQ2 trajectories with and without the antigen present clearly shows a rigidification of the conformational space upon antigen binding and thus follows the paradigm of conformational selection.

**Figure 6:**
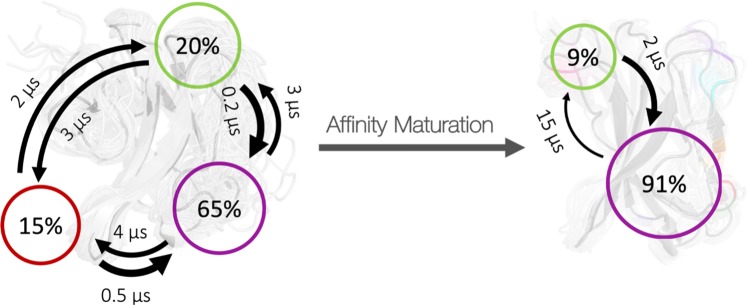
Markov-state model with respective state probabilities and transition timescales. In the background the substantial broader ensemble of conformations for the wildtype 1BEC (left) compared to the affinity matured 2APX (right) is illustrated.

Based on the observed decrease in conformational diversity depending on the presence of the antigen SEC 3, the 2APF/2AQ2 variants clearly follows the paradigm of conformational selection and the resulting population shift upon binding is visualized in Fig. 5. Figure 5 illustrates the resulting conformational ensembles of the hierarchical clustering of the 8.8 µs trajectories of the 2APF and the 1 µs trajectories of the 2AQ2 of the CDR 2 and HV 4 loops by aligning on the whole TCR ß variable domain. This clustering led to 14 clusters for the 2APF and to 4 clusters for the 2AQ2 bound to SEC 3. Figure 6 provides kinetic characterization of conformational transitions and results in short transition timescales for the wildtype 1BEC TCR Vß variant, while it reveals slightly longer timescales in the higher microsecond timescale for the matured 2APX variant. The Markov-state model of the 2APX TCR Vß variant reveals one dominantly populated state, while the 1BEC has three probable states with fast exchanges between these macrostates.

**Figure 7:**
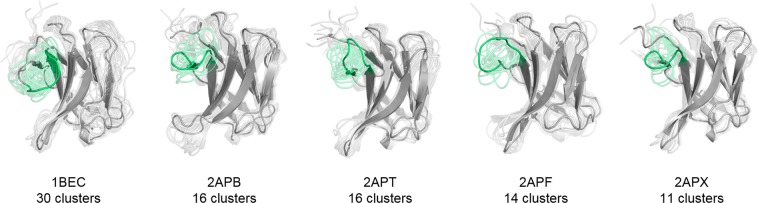
CDR 3 loop ensemble of the hierarchical clustering of the 24.1 µs 1BEC trajectories, the 9.5 µs 2APB trajectories, the 7.0 µs 2APT trajectories, the 8.8 µs 2APF trajectories and the 4.8 µs 2APX trajectories. Even though the CDR 3 loop is not directly involved in binding it is still rigidified during affinity maturation.

In the systems studied CDR 2 and HV 4 are the major loops binding the antigen, thus we primarily focused on these two loops. Surprisingly, the CDR 3 loop, even though not directly involved in binding to SEC 3, still is significantly rigidified during affinity maturation. Figure 7 illustrates the resulting conformational ensembles of the CDR 3 loop by applying the hierarchical clustering with a distance cut-off of 2 Å and reveals a significant decrease in conformational diversity in different stages of affinity maturation.

## Discussion

This present study characterizes the conformational diversity during affinity maturation of the CDR 2 and HV 4 loops, because of their strong involvement in antigen binding. Strong experimental structural information together with molecular dynamics simulations allow the investigation of conformational selection, the consequences of point-mutations on flexibility and changes in kinetics and thermodynamics during this affinity maturation pathway.

The concept of conformational selection suggests that within this pre-existing ensemble of conformations the binding competent state is selected, accompanied by a population shift and redistribution of the conformational states^20,24,25^. The theory of having an ensemble of pre-existing conformations out of which the functional ones are selected was suggested Pauling^25,26^ and demonstrated by Milstein and Wedemayr^27,28^. This view has been supported by the population shift or conformational selection model, originated from the Monod–Wyman–Changeux model^29^. This new view on proteins, i.e., that one sequence can show high structural diversity, facilitated the understanding and evolution of new functions and structures^21^. Proper characterization of the CDR loops, especially the loops which are mainly involved in the binding process, is crucial to understand protein-protein interactions and antigen binding^30,31^. Figure 2 compares the intramolecular interactions of three variants in different stages of affinity maturation. While there is no difference in intramolecular stabilizing hydrogen bond or ionic interactions in the binding site between the wildtype and a further matured variant 2APF the most matured TCR Vß variant 2APX forms a significantly higher number of hydrogen bonds and ionic interactions. This indicates a substantial stabilization on a static structural level. The mutations in the CDR 2 and HV 4 loops are in line with the observed rigidification (Fig. 3) of these loops upon affinity maturation, which are directly involved in binding to SEC 3. Also the CDR 3 loop without showing interactions with the antigen SEC 3 reveals as a consequence of mutations a rigidification upon affinity maturation. Various studies already focused on elucidating the affinity maturation process and the numerous diverse involved mechanisms, including improved shape complementarity in the binding site, an increase in buried surface area upon complex formation, additional interfacial polar or hydrophobic interactions and rigidification of the binding site^18,28,32–35^. Figure 3 shows an overview of all simulated variants with their resulting number of cluster representatives by using the same distance cut-off. The number of resulting clusters is one possible way to characterize flexibility and reveals a substantial rigidification especially when comparing the most matured variant with the wildtype. Another possibility to compare the flexibility among the variants is to take the conformational space into account. We used tICA coordinates, because we were interested in retaining the kinetics of exchanges between conformational sub-states occurring on different timescales. Figure 4 shows tICA plots of five exemplary variants in distinct maturation stages using the same coordinate system in combination with a representative ensemble of CDR 2 and HV 4 loop structures, including colored point-mutations corresponding to Fig. 1. The conformational diversity of the representative ensembles in Fig. 4 decreases significantly from wildtype to the most matured variant. Also, the conformational space of the matured 2APX variant is substantially reduced compared to the wildtype 1BEC. Additionally, a clear population shift can be observed throughout all considered variants during this maturation pathway. The available X-ray structures, crystallized with and without antigen, are located in the same local side minimum, which becomes the dominant minimum in solution of the most matured 2APX variant. The resulting conformational selection is illustrated in Fig. 5. We clearly see a strong population shift of the 2APF variant to the bound 2AQ2 and a significantly decreased conformational space of the CDR 2 and HV 4 loops if the antigen SEC 3 is present. Figure 6 characterizes the transition timescales of sub-states of the accessible conformational space and the resulting kinetics reveal fast transition timescales for the wildtype 1BEC, while the matured 2APX displays slower transition kinetics. This indicates that the rigidification shifts the probability toward a small number of states and thereby increases specificity. The probabilities of the most matured 2APX variant are shifted towards the binding competent state, while the wildtype 1BEC has higher probabilities for various other sub-states which are accessible in the nanosecond timescale. In this affinity maturation study the CDR 3 loop is not involved in the antigen-binding process, but it still remains one of the most challenging loops in terms of structure prediction and characterization in solution^36^. Therefore we used our long-timescale simulations to analyze the conformational diversity of the CDR 3 loop in solution. Figure 7 reveals a decrease in flexibility of the CDR 3 loop during the affinity maturation pathway reflected in the reduced number of clusters.

## Methods

A previously published method characterizing the CDR-H3 loop ensemble upon antigen binding in solution^12,22^ was used to investigate the conformational diversity of CDR 2 and HV 4 loops of TCR Vß variants in different stages of affinity maturation. Experimental structure information was available for all stages of affinity maturation and we used all available crystal structures as starting structures for molecular dynamics simulations. The TCR Vß variant with the PDB accession code 2AQ2 was simulated with the antigen SEC 3 bound. All other available complex structures were simulated without the presence of the antigen. The starting structures for simulations were prepared in MOE (Molecular Operating Environment, Chemical Computing Group, version 2018.01) using the Protonate3D tool^37,38^. To neutralize the charges we used the uniform background charge^39–41^. Using the tleap tool of the AmberTools16^39,40^ package, the crystal structures were soaked with cubic water boxes of TIP3P water molecules with a minimum wall distance of 10 Å to the protein^42^. For all crystal structures parameters of the AMBER force field 14SB were used^43^. The TCR Vß variants were carefully equilibrated using a multistep equilibration protocol^44^.

### Metadynamics simulations

To enhance the sampling of the conformational space well-tempered metadynamics^45–47^ simulations were performed in GROMACS^48,49^ with the PLUMED 2 implementation^50^. As collective variables, we used a linear combination of sine and cosine of the ψ torsion angles of the CDR 2 loop and the HV 4 loop calculated with functions MATHEVAL and COMBINE implemented in PLUMED 2^50^. As discussed previously the ψ torsion angle captures conformational transitions comprehensively^37,38^. The decision to additionally include the HV 4 loop ψ torsion angles is based on the strong involvement of the HV 4 loop in the binding to SEC 3 as evident from the X-ray structure of the complex. The simulations were performed at 300 K in an NpT ensemble. The height of the Gaussian was determined according to minimal distortion of the TCR systems, resulting in a Gaussian height of 10.0 kcal/mol. Gaussian deposition occurred every 1000 steps and a biasfactor of 10 was used. 1 µs metadynamics simulations were performed for each available TCR Vß variant crystal structure. The resulting trajectories were clustered in cpptraj^40,51^ by using the average linkage hierarchical clustering algorithm with a distance cut-off criterion of 1.2 Å resulting in a large number of clusters. The cluster representatives for the TCR Vß variants in different stages of affinity maturation with the PDB accession codes 1BEC, 2APB, 2APT, 2APF, 2APX without the antigen present and 2AQ2 with the antigen present were equilibrated and simulated for 100 ns using the AMBER16^39^ simulation package.

### Molecular dynamics simulations

Molecular dynamics simulations were performed in an NpT ensemble using pmemd.cuda^52^. Bonds involving hydrogen atoms were restrained by applying the SHAKE algorithm^53^, allowing a time step of 2.0 fs. Atmospheric pressure of the system was preserved by weak coupling to an external bath using the Berendsen algorithm^54^. The Langevin thermostat^55^ was used to maintain the temperature during simulations at 300 K.

For the resulting trajectories cluster analyses were performed on the CDR 2 and HV 4 loop using the hierarchical clustering algorithm implemented in cpptraj^40^ with a distance cut-off criterion of 2 Å to get a representative ensemble of structures. Additionally, a time-lagged independent component analysis (tICA) was performed using the python library PyEMMA 2 employing a lag time of 10 ns^56^. Thermodynamics and kinetics were calculated with a Markov-state model^57^ by using PyEMMA 2, which uses the k-means clustering algorithm^58^ to define microstates and the PCCA+ clustering algorithm^59^ to coarse grain the microstates to macrostates. PCCA+ is a spectral clustering method, which discretizes the sampled conformational space based on the eigenvectors of the transition matrix. The sampling efficiency and the reliability of the Markov-state model (e.g., defining optimal feature mappings) can be evaluated with the Chapman-Kolmogorov test^60,61^, by using the variational approach for Markov processes^62^ and by taking into account the fraction of states used, as the network states must be fully connected to calculate probabilities of transitions and the relative equilibrium probabilities. To build the Markov-state model we used the backbone torsions of the CDR 2 and the HV 4 loops, defined 150 microstates using the k-means clustering algorithm and applied a lag time of 10 ns.

The images presented in this paper were created by using the PyMOL molecular graphics system^63^.

## Conclusion

To elucidate the mechanism of affinity maturation we analyzed a series of TCR Vß variants with strong experimental structural information in different stages of affinity maturation. Besides the structural intramolecular stabilization, we observed a substantial reduction in flexibility and resulting conformational diversity of the CDR 2 and HV 4 loop, which are directly involved in antigen binding. The investigated variants follow the conformational selection paradigm accompanied by a significant population shift towards the binding competent conformation in the further matured 2APF variant. We characterized the transition kinetics between different conformational states and observed a conformational shift during this affinity maturation pathway towards the binding competent state in the matured 2APX variant. Our results clearly reveal a rigidification and a strong population shift during the affinity maturation process.

## Supplementary information

Supplementary Information.
