# Supplementary material for: T-Cell Receptor Variable β Domains Rigidify During Affinity Maturation
Source: Sci Rep. 2020 Mar 11;10:4472. doi: 10.1038/s41598-020-61433-0 (PMC7066139; doi:10.1038/s41598-020-61433-0)
Supplement: Supplementary file 1 — Supplementary Information. [file 41598_2020_61433_MOESM1_ESM.pdf]

Supplemental Information

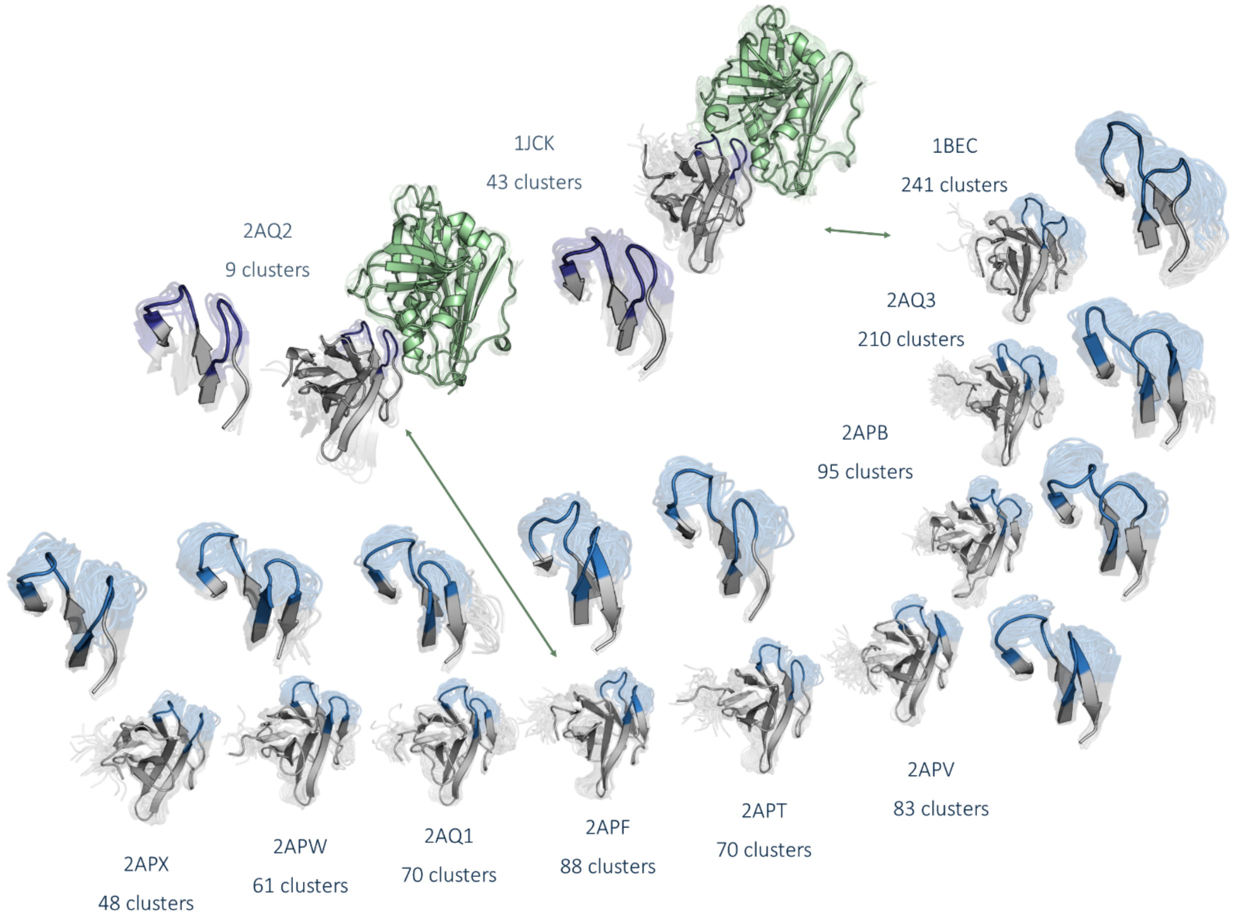

SI Figure S1: *Overview of all analyzed TCR variants with the resulting conformational ensembles of the CDR 2 and the HV 4 loop colored in blue of each 1 µs metadynamics simulations. The diversity of the conformational ensemble is characterized by the number of clusters for each simulation and decreases with higher stages of maturation. The variants 1JCK and 2AQ2 were additionally simulated with the antigen present to investigate the role of the SEC3 on the observed conformational ensemble. The resulting conformational ensembles of these simulations follow the paradigm of conformational selection.*
